# Reflective Engagement With a Digital Physical Activity Intervention Among People Living With and Beyond Breast Cancer: Mixed Methods Study

**DOI:** 10.2196/51057

**Published:** 2024-02-09

**Authors:** Michael C Robertson, Emily Cox-Martin, Karen Basen-Engquist, Elizabeth J Lyons

**Affiliations:** 1 Department of Family and Preventive Medicine TSET Health Promotion Research Center University of Oklahoma Health Sciences Center Oklahoma City, OK United States; 2 Department of Nutrition, Metabolism & Rehabilitation Sciences University of Texas Medical Branch at Galveston Galveston, TX United States; 3 VA Puget Sound Health Care System Tacoma, WA United States; 4 Department of Health Disparities Research University of Texas MD Anderson Cancer Center Houston, TX United States

**Keywords:** survivors of cancer, exercise, acceptance and commitment therapy, fatigue, mindfulness, motivation, behavioral sciences

## Abstract

**Background:**

People living with and beyond breast cancer can face internal barriers to physical activity (eg, fatigue and pain). Digital interventions that promote psychological acceptance and motivation may help this population navigate these barriers. The degree to which individuals (1) adhere to intervention protocols and (2) reflect on and internalize intervention content may predict intervention efficacy.

**Objective:**

The objective of this study was to characterize the nature of reflective processes brought about by an 8-week acceptance- and mindfulness-based physical activity intervention for insufficiently active survivors of breast cancer (n=75). Furthermore, we explored the potential utility of a metric of reflective processes for predicting study outcomes.

**Methods:**

Of the intervention’s 8 weekly modules, 7 (88%) included an item that asked participants to reflect on what they found to be most useful. Two coders conducted directed content analysis on participants’ written responses. They assessed each comment’s depth of reflection using an existing framework (ranging from 0 to 4, with 0=*simple description* and 4=*fundamental change with consideration of social and ethical issues*). The coders identified themes within the various levels of reflection. We fit multiple linear regression models to evaluate whether participants’ (1) intervention adherence (ie, number of modules completed) and (2) the mean level of the depth of reflection predicted study outcomes.

**Results:**

Participants were aged on average 57.2 (SD 11.2) years, mostly non-Hispanic White (58/75, 77%), and mostly overweight or obese (54/75, 72%). Of the 407 responses to the item prompting personal reflection, 70 (17.2%) were rated as reflection level 0 (ie, *description*), 247 (60.7%) were level 1 (ie, *reflective description*), 74 (18.2%) were level 2 (ie, *dialogic reflection*), 14 (3.4%) were level 3 (ie, *transformative reflection*), and 2 (0.5%) were level 4 (ie, *critical reflection*). Lower levels of reflection were characterized by the acquisition of knowledge or expressing intentions. Higher levels were characterized by personal insight, commentary on behavior change processes, and a change of perspective. Intervention adherence was associated with increases in self-reported weekly bouts of muscle-strengthening exercise (B=0.26, SE 0.12, 95% CI 0.02-0.50) and decreases in sleep disturbance (B=−1.04, SE 0.50, 95% CI −0.06 to −2.02). The mean level of reflection was associated with increases in psychological acceptance (B=3.42, SE 1.70, 95% CI 0.09-6.75) and motivation for physical activity (ie, integrated regulation: B=0.55, SE 0.25, 95% CI 0.06-1.04).

**Conclusions:**

We identified a useful method for understanding the reflective processes that can occur during digital behavior change interventions serving people living with and beyond breast cancer. Intervention adherence and the depth of reflection each predicted changes in study outcomes. Deeper reflection on intervention content was associated with beneficial changes in the determinants of sustained behavior change. More research is needed to investigate the relations among digital behavior change intervention use, psychological processes, and intervention efficacy.

## Introduction

### Background

There were an estimated 4.1 million people living with and beyond breast cancer in 2022 [1]. This number is estimated to reach nearly 5 million by 2030 [2]. This population can encounter long-term challenges related to health and quality of life, including fatigue, anxiety and depressive symptoms, breast cancer–related lymphedema, metabolic dysregulation, bone loss and osteoporosis, and cancer recurrence [3,4]. Physical activity may protect people living with and beyond breast cancer from these problems [5-7]. However, most people who have been diagnosed with breast cancer do not meet nationally recommended physical activity guidelines [8,9].

Psychotherapy-informed digitally delivered acceptance- and mindfulness-based approaches may facilitate physical activity promotion for people living with and beyond breast cancer. People living with and beyond breast cancer commonly cite uncertainty, frustration, cancer-related fatigue, and pain to be barriers to physical activity [10-13]. Interventions that can help people to better navigate the uncomfortable thoughts and sensations that can act as impediments to physical activity may support their efforts to be more physically active. Authoritative entities recommend acceptance- and mindfulness-based interventions for people living with and beyond cancer; strong evidence supports their efficacy for reducing anxiety and depressive symptoms in this population, and they have been shown to reduce cancer-related fatigue [14-17]. Furthermore, physical activity interventions that simultaneously target both health-related behaviors and quality-of-life issues are more effective at achieving sustained physical activity outcomes than interventions that only promote physical activity for this population [18]. Emerging evidence suggests that acceptance- and mindfulness-based physical activity interventions can be feasible and effective at promoting physical activity [19-21] and that digitally delivered approaches can be acceptable and potentially effective for people living with and beyond cancer [22,23].

The degree to which participants meaningfully engage with and internalize health promotion content may determine the efficacy of digital behavior change interventions (DBCIs). However, achieving high levels of engagement can be a marked challenge for DBCIs [24]. Participants often exhibit low adherence to digital physical activity interventions and poor study retention [25]. Accordingly, researchers have tended to prioritize measuring and optimizing intervention use (eg, optimizing metrics such as the number of modules completed and time spent in an app) [26]. However, using system use data alone as a proxy for engagement is problematic. First, the relationship between these metrics and intervention efficacy is not always straightforward. Although there is sometimes a positive linear relationship between DBCI use and intervention efficacy, this is not always the case [25,27,28]. DBCIs may tend to have certain thresholds of engagement that confer the majority of the benefits, or intervention components may interact with one another. Furthermore, engagement with a DBCI may be expected to taper over time and even be supplanted by engaging in the desired behavior itself [29]. Second, focusing exclusively on quantitative use metrics affords only limited insight into the psychological aspects underpinning participants’ experiences with DBCIs. This precludes achieving a full understanding of the DBCI’s mechanisms of action. Thus, researchers have called for broadening our conceptualization of DBCI engagement [28].

A growing literature highlights the importance of understanding the psychological processes that occur when participants engage with DBCI content; this includes supplementing system use metrics with additional aspects of DBCI engagement that pertain to affect, attention, interest, immersion, flow, and reflection [26,29,30]. Researchers of human-computer interaction have discussed engagement in similar terms, parsing engagement according to notions of behavioral adherence, behavioral effort (eg, discussing emotions, thoughts, and behaviors in intervention), cognition, and affect [31]. Researchers with an educational perspective have provided similar definitions of engagement with digital systems, noting that the cognitive and affective aspects of engagement are largely neglected [32]. Yang et al [25] have contributed a framework specific to physical activity promotion in mobile health in which they conceptualize engagement as being determined by breadth, depth, interaction, and length of engagement. These broad conceptualizations are useful for helping to orient researchers toward the many and varied facets of engagement. Rather than attempting to establish a universal conceptualization, it may be advantageous to tailor the conceptualizations of engagement based on context [33]. Using qualitative and mixed methods to investigate the nature of participants’ engagement with DBCIs may be particularly useful to this end [24,26,29].

*Reflection* is an essential process for self-improvement and making lasting behavior changes in the context of DBCIs [34]. As applied to health-related education, reflection and reflective processes connotate deliberate critical analysis of knowledge and experiences to achieve a fuller understanding [35]. The process of reflecting on the outcomes of past and ongoing efforts or newly acquired information helps individuals integrate knowledge and skills into practice and overcome persistent barriers to behavior change [35,36]. Reflection may be particularly relevant in the context of DBCIs because it may allow people to gain meaningful insights from their personal health-related data that can support lasting behavior change [34]. In the education literature, reflective thinking and writing are commonly used strategies to prompt personal reflection, and it is a common practice to evaluate written responses to determine to what degree an individual has considered and applied didactic content in the context of their own lived experiences [37-39]. Fleck and Fitzpatrick [40] present a framework that operationally defines 5 levels of reflection. This framework has been shown to be particularly useful for evaluating the levels of reflection promoted by the design features of DBCIs [34]. In this study, we sought to extend this literature by characterizing the reflective processes engaged in by people living with and beyond cancer who experienced a digital acceptance- and mindfulness-based physical activity intervention.

### Objectives

It may be possible to help people living with and beyond breast cancer reframe some of the unpleasant internal sensations that can act as barriers to physical activity (eg, pain, fatigue, and frustration). Understanding the degree to which participants reflect on and internalize digital intervention content targeting these psychological processes may be useful for understanding how best to support this population. This study answers calls to investigate the deeper psychological aspects involved in engagement with DBCIs to provide insight into the interplay among intervention use, individual experiences, and intervention efficacy. The aims of this study were to (1) characterize the nature of reflective processes brought about by a DBCI designed to increase physical activity in insufficiently active people living with and beyond breast cancer and (2) explore the potential utility of a metric of reflective processes in DBCIs within an acceptance- and mindfulness-based physical activity intervention.

## Methods

### Study Design

We conducted a secondary analysis on data obtained from a 1-group pilot study. The purpose of the parent study was to evaluate the acceptability of an acceptance- and mindfulness-based intervention to increase physical activity in survivors of breast cancer [23]. This study is an investigation of the use of the intervention. Participants were female adults (aged ≥18 y) who had been diagnosed with breast cancer but were not actively preparing for surgery or undergoing chemotherapy or irradiation treatment (n=75). Eligibility criteria included that participants reported engaging in <150 minutes of moderate-intensity aerobic physical activity per week. We recruited participants using a large listserve of individuals who were interested in receiving information about breast cancer–related research studies. Study staff contacted interested individuals via telephone to assess eligibility.

### Ethical Considerations

Study procedures were approved by the University of Texas School of Public Health Committee for the Protection of Human Subjects (HSC-SPH-18-1025). All participants provided informed consent for participation.

### Intervention Description

The intervention has been described in detail elsewhere [23]. Briefly, the ACTive program was an 8-week DBCI designed to help insufficiently active survivors of breast cancer increase moderate-intensity aerobic and muscle-strengthening physical activity. It was centered on increasing physical activity acceptance (ie, cognitive acceptance and behavioral commitment) and autonomous motivation for physical activity (ie, enjoyment, values, interest, and identification). It was grounded in acceptance and commitment therapy (ACT) principles (ie, values, committed action, acceptance, defusion, and contacting the present moment). It consisted of 8 modules (along with a brief introductory module), each of which was delivered electronically via a weekly REDCap (Research Electronic Data Capture; Vanderbilt University) survey. The modules included didactic content and experiential exercises targeting core ACT principles. They were presented in brief videos, audio files, images, and other documents. Participants were prompted to input information periodically (eg, physical activity levels and reactions and thoughts on experiential exercises). Branching logic within REDCap was used to remind participants of their responses to items from previous modules as well as provide feedback on their responses within modules (eg, an emoji appeared if responses indicated that the participant had met her physical activity goal for the week). The modules contained a repository of additional optional content (eg, muscle-strengthening physical activity videos for survivors of cancer, videos with yoga classes for survivors of breast cancer, and a video on proper walking posture for survivors of cancer).

At the end of modules 1 to 7, an item asked participants, “What is one important, personal take-away point from this session?” This item was included to identify specific aspects of the intervention that were perceived as the most *useful* by participants because usefulness is an important component of acceptability. Participants were presented with an open-text box to provide a reply. Providing participants with the opportunity to reply to open-ended items such as this can help contribute to a more in-depth understanding of participants’ engagement and experience with DBCI content [26].

### Measures

#### Physical Activity Behaviors

The Godin Leisure-Time Exercise Questionnaire was administered to obtain pre- and postintervention estimates of participants’ average weekly leisure time aerobic physical activity over the past month [41,42]. To obtain estimates of participants’ muscle-strengthening physical activity levels, we included an item derived from the Godin Leisure-Time Exercise Questionnaire [43,44]. This item asked, “In a typical week, outside of your job or work around the house, how many days do you do leisure-time physical activities specifically designed to strengthen your muscles such as lifting weights, circuit training, or resistance bands? (Do not include cardio/aerobic types of exercise).” Response options ranged from 0 to 7.

#### Physical Activity Acceptance

The ACTive program was centered on increasing physical activity acceptance. This was defined as one’s willingness to experience the negative internal experiences that can sometimes be associated with physical activity, rather than avoiding them. This was operationalized by the Physical Activity Acceptance Questionnaire (PAAQ) [45]. The PAAQ consists of two 5-item subscales. The *cognitive acceptance* subscale measures one’s propensity to accept the reality of unpleasant sensations associated with physical activity, whereas the *behavioral commitment* subscale pertains to persisting in committed action despite the occurrence of challenging thoughts or sensations. Responses on items of the PAAQ range from 1=*never true* to 7=*always true*. For scoring, the items on the *cognitive acceptance* subscale are reverse coded, and the items of each subscale are summed (range 5-35). This questionnaire was administered before and after the intervention.

#### Motivation for Physical Activity

The ACTive program also aimed to increase motivation for physical activity as conceptualized by self-determination theory (SDT). SDT parses motivation conceptually based on the degree to which it is autonomous in nature. SDT posits that changes in more autonomous motivations (eg, integrating a behavior into relevant self-narratives) will yield longer-lasting behavior changes than changes in less autonomous motivations (eg, receiving a *badge* as reinforcement for performance). We operationalized motivation for physical activity before and after the intervention using the Behavioral Regulation for Exercise Questionnaire-3 (BREQ-3). The BREQ-3 consists of six 4-item subscales (*amotivation*, *external regulation*, *introjected regulation*, *identified regulation*, *integrated regulation*, and *intrinsic regulation*). The *intrinsic regulation* subscale captures a highly autonomous form of motivation, defined by the degree to which one engages in a behavior because one finds it inherently interesting or enjoyable. The *integrated regulation* subscale captures another highly autonomous form of motivation, an extrinsic form of motivation defined by the degree to which an individual has fully internalized the reason for action owing to finding it concordant with their values. The *identified regulation* subscale captures a somewhat less autonomous form of motivation (although still relatively autonomous overall), defined by the degree to which an individual consciously values a reason for action. For scoring, the mean scores for each set of items are calculated (range 0-4).

#### Health-Related Outcomes

For exploratory purposes, we administered measures of quality of life and physical functioning before and after the intervention. To operationalize these constructs, we used the National Institutes of Health–funded Patient-Reported Outcomes Measurement Information System (PROMIS)-29 profile measure (version 2.1) [42]. The PROMIS-29 consists of 8 subscales: *physical function* (eg, “Are you able to run errands and shop?”), *anxiety* (eg, “In the past 7 days...I felt fearful”), *depressive symptoms* (eg, “In the past 7 days...I felt worthless”), *fatigue* (eg, “In the past 7 days...I felt fatigued”), *sleep disturbance* (eg, “In the past 7 days...I had a problem with my sleep”), *ability to participate in social roles and activities* (eg, “I have trouble doing all of my regular leisure activities with others”), *pain interference* (eg, “In the past 7 days...How much did pain interfere with your day to day activities?”), and *pain intensity* (eg, “How would you rate your pain on average?”). All subscales except the *pain intensity* subscale have 4 items and 5-point Likert-type responses ranging from 0 (*not at all*) to 4 (*very much*). The *pain intensity* subscale has 1 item and an 11-point scale ranging from 0 (*no pain*) to 10 (*worst pain imaginable*). Scores are coded, summed, and converted to T-scores such that higher scores indicate more of the concept being measured (eg, range for *physical functioning* subscale: 22.5-57.0).

### Reflection Framework

Reflection levels were based on the reflection framework presented by Fleck and Fitzpatrick [40]. This framework emerged from research conducted in the context of the design of digital technologies and human-computer interaction. It defines 5 levels of reflection intended to serve as a resource for thinking about, and designing for, reflection. Reflection level 0 (revisiting) is defined as “Description or statement about events without further elaboration or explanation. Not reflective*.*” Reflection level 1 (reflective description) is defined as “Description including justification or reasons for action or interpretation, but in a reporting or descriptive way. No alternate explanations explored, limited analysis and no change of perspective*.*” Reflection level 2 (dialogic reflection: exploring relationships) is defined as “A different level of thinking about intervention content. Identifying or exploring relationships between relevant concepts. Applying experience or knowledge, providing evidence of cycles of interpreting and questioning, consideration of different explanations, hypotheses and/or other points of view*.*” Reflection level 3 (transformative reflection: fundamental change) is defined as “Revisiting an event or knowledge with intent to re-organize and/or do something differently. Asking fundamental questions and challenging personal assumptions leading to a change in practice or understanding*.*” Finally, reflection level 4 (critical reflection: wider implications) is defined as “Social and ethical issues are taken into consideration. Generally considering the (much wider) picture*.*”

### Data Analysis

#### Qualitative Data Analysis

MCR and EJL conducted 2 phases of directed content analysis on participants’ written responses to the open-ended item asking, “What is one important, personal take-away point from this session?” [46]. First, the coders independently rated the reflection level of each individual response by evaluating the response against the reflection framework presented by Fleck and Fitzpatrick [40]. Throughout this process, they produced descriptors to extend and apply the definitions presented by Fleck and Fitzpatrick [40] of the various reflection levels to our study context. The 2 coders first coded all the responses from 1 module independently. Next, they met to discuss the functional definitions and descriptors of reflection levels in our study context and resolve coding discrepancies. The coders then evaluated the rest of the responses independently. After doing so, they met to reconcile discrepancies. The coders created a table, based on the reflection framework presented by Fleck and Fitzpatrick [40] as well as supplemental descriptors and illustrative examples from this study, to further clarify their conception of the various reflective levels as they might be applied in the context of DBCIs. For the second phase of directed content analysis, MCR sorted responses by reflection level and MCR and EJL coded responses using inductive codes that were informed by principles of behavioral science (eg, behavior change techniques) and ACT [46]. MCR coded all responses first and provided a list of inductive codes to EJL. Next, EJL coded all responses using the list of inductive codes provided by MCR and adding additional codes as needed. The 2 coders then met to discuss codes and reconcile differences.

#### Quantitative Data Analysis

We computed descriptive statistics for participant demographics, study outcome variables, intervention adherence, and each participant’s mean level of reflection (ie, each individual’s mean score of the items scored via the qualitative procedures detailed in the previous subsection). We defined intervention adherence as the number of modules that each participant completed; this is a commonly used measure of engagement with DBCIs [26]. We then conducted multiple linear regression analyses with maximum likelihood estimation. The independent variables were (1) the number of modules completed and (2) participants’ mean level of reflection. The dependent variables (in separate models) were the follow-up measures of self-reported aerobic and muscle-strengthening physical activity, the PAAQ, the BREQ-3, and the PROMIS-29 subscales described previously. All analyses adjusted for the baseline value of the dependent variable and, given our interest in parsing the possible effects of the breadth versus the depth of engagement, the other independent variable of interest (ie, both intervention adherence and the mean level of reflection were included in all models). All analyses also adjusted for sociodemographic and cancer-related factors that we identified a priori as potentially confounding variables. Specifically, we adjusted for age (years), education level (no bachelor’s degree, bachelor’s degree, or graduate school), ethnicity (Hispanic or not Hispanic), race (American Indian or Alaska Native or other, Asian, Black or African American, or White), BMI category (underweight, normal, overweight, or obese), time since cancer diagnosis (years), and stage at diagnosis (1, 2, or 3/4). Missing data were handled using full information maximum likelihood. We set our nominal α level to .05 for all analyses. All statistical analyses were performed using R (version 4.0.3; R Foundation for Statistical Computing).

## Results

### Participant Demographic Characteristics

The average age of the participants was 57.2 (SD 11.2; range 31-78) years. The median time since breast cancer diagnosis was 8.2 (IQR 3-12) years. The participants were relatively well educated, mostly non-Hispanic White (58/75, 77%), and mostly overweight or obese (54/75, 72%; Table 1).

**Table 1:** Participant characteristics.

Characteristic and category			Participants, n (%)
**Education level (n=75)**			
	High school diploma or GED^a^	0 (0)	
	Some college	16 (21)	
	Bachelor’s degree	34 (45)	
	Graduate school degree	25 (33)	
**Stage of breast cancer at diagnosis (n=71)**			
	1	32 (45)	
	2	28 (39)	
	3	9 (13)	
	4	2 (3)	
**Race (n=75)**			
	American Indian, Alaska Native, or other	1 (1)	
	Asian	4 (5)	
	Black or African American	7 (9)	
	White	63 (84)	
**Ethnicity (n=74)**			
	Hispanic	7 (10)	
	Non-Hispanic	67 (91)	
**Marital status (n=74)**			
	Single	11 (15)	
	Married	54 (73)	
	Living with significant other	1 (1)	
	Divorced	5 (7)	
	Widowed	3 (4)	
**Employment status (n=68)**			
	Employed full-time	38 (56)	
	Employed part-time	10 (15)	
	Retired	20 (29)	
**BMI status (n=74)**			
	Underweight	1 (1)	
	Normal	19 (26)	
	Overweight	32 (43)	
	Obese	22 (30)	

^a^GED: General Educational Development Test.

### Intervention Adherence

The median number of the 8 modules completed was 8 and the first quartile value was 4 modules (IQR 4). The minimum number of modules completed was 1 (7/75, 9%).

### Coding the Levels of Reflection

The coders created a table while going through the process of coding the reflections based on the reflection framework presented by Fleck and Fitzpatrick [40]. We added descriptors and illustrative examples from this study to further clarify our conception of the various reflective levels as they might be applied in the context of DBCIs (Table 2).

**Table 2:** Levels of reflection with relevant descriptors and illustrative examples.

Reflection level^a^ and additional descriptors		Illustrative examples
**Reflection level 0. Description: revisiting (description or statement about events without further elaboration or explanation; not reflective)**		
	Repeating or paraphrasing intervention content	“That exercise makes you less tired but also helps you sleep Better.” [SID^b^ 157, 47 years]
	Superficial imperative statements	“[G]et up and move.” [SID 95, 49 years]
	Surface-level comments	“I thought the extended mindfulness video was helpful.” [SID 100, 37 years]
	Platitudes	“Life is hard sometimes.” [SID 99, 68 years]
	Not responsive to the prompt	“Bad weather.” [SID 117, 64 years]
**Reflection level 1. Reflective description: revisiting with explanation (description including justification or reasons for action or interpretation but in a reporting or descriptive way; no alternate explanations explored, limited analysis, and no change of perspective)**		
	Elaborating upon intervention content	“On our sickest days, there is more going right in our bodies than going wrong. I need to appreciate this. And celebrate this.” [SID 113, 50 years] “[T]hat the uncomfortable feel of exercise is actually good for me. I just need to embrace it.” [SID 140, 60 years]
	Imperative statements (including justification or reasons or descriptive strategy)	“I need to get moving, so I will feel better.” [SID 79, age not given]
	Personal insight	“[T]hat I’m good at putting things off.” [SID 134, 60 years] “I am totally the kid looking out the window—I try to make everything an adventure and to look at the positive mental attitude.” [SID 103, 45 years]
	Skill building or learning a technique	“I’ve learned how to focus away from the chatter in my brain.” [SID 99, 68 years] “[T]hat I can ‘pick up’ internal barriers which relieves some of the feeling of frustration and feeling like a failure.” [SID 98, 53 years] “I liked the shear stress explanation. I can picture that while I exercise.” [SID 100, 37 years]
**Reflection level 2. Dialogic reflection: exploring relationships (a different level of thinking about intervention content; identifying or exploring relationships between relevant concepts; applying experience or knowledge, providing evidence of cycles of interpreting and questioning, and consideration of different explanations, hypotheses, and other points of view)**		
	Applying intervention content to one’s own life	“My old excuse of saying ‘I just don’t want to exercise,’ is not a good enough reason. I know the reasons are that it’s uncomfortable and inconvenient, but that’s really not true. Also, I know I can adapt and it won’t cause pain in my hip with bursitis.” [SID 145, 48 years]
	Commentary on the nature of the relationships between disparate concepts	“If I am going to change my fitness habits, I must see how they relate to my values.” [SID 124, 42 years]
	Taking a different perspective	“[T]hat I’m being invited, not required to experiment with activity and that I can choose how to do it. I liked the line about experimenting with what we’re being told the benefits are in our own bodies. That makes me feel more in control and interested.” [SID 137, 53 years]
	Applying new skills or knowledge and reflecting on this	“In the almost 14-minute extended mindfulness exercise, I found that it did relax me even though that wasn’t the objective. I kept my eyes closed during the entire exercise. At first, my other thoughts included anxiety over today’s election. One of the suggestions was to look at yourself from outside yourself. I find I’m usually able to do that anyway...as it enables me to be more compassionate and understanding of others’ pain because of what I’ve experienced. For me, the only distraction, as I kept my eyes closed, was hearing the/your voice telling me things. I had no problem with pushing away thoughts because the only thing I was seeing was inside my eyes as they were closed. Once opened, everything becomes a distraction.” [SID 133, 70 years]
**Reflection level 3. Transformative reflection: fundamental change (revisiting an event or knowledge with intent to reorganize and do something differently; asking fundamental questions and challenging personal assumptions leading to a change in practice or understanding)**		
	Perspective significantly altered	“[T]hat I and many other people get very wrapped up in goals and disguise them as values. We find that we’re not measuring up and we get disappointed in ourselves and give up working at them. Looking at goals as finite and values as infinite and guiding principles that shape goals puts them in a different perspective. It gives me hope that I won’t judge myself too harshly if I don’t fulfill my goals, that I’ll realize that maybe that goal wasn’t right for me and didn’t fit in well with my values.” [SID 137, 53 years]
	A change in practice or understanding resulting from personal insight	“I am stronger than the spoiled, damaged, hurt child inside me. I don’t have to listen to her. Just put her plump pouty face in my backpack and carry on.” [SID 82, 64 years]
**Reflection level 4. Critical reflection: wider implications (social and ethical issues are taken into consideration; generally considering the wider picture)**		
	A transformative reflection that weaves in broader social or ethical considerations	“My essential Self is still there...I am alive, the tiny kernel of me, the spark, though almost extinguished, has to be nurtured above all else now...or I am extinguished by the grotesque cancer industry conveyor belt.” [SID 110, 74 years]

^a^From Fleck and Fitzpatrick [40].

^b^SID: study identification number.

### Levels of Reflection

#### Overall

There were 407 total responses from the 75 participants over the course of the 7 modules that featured the open-ended item for reflection (the item was not included in module 8). Participants submitted an average of 5.4 (SD 2.2) out of 7 possible responses. Of the 407 responses, we rated 70 (17.2%) as reflection level 0 (ie, *description*), 247 (60.7%) as level 1 (ie, *reflective description*), 74 (18.2%) as level 2 (ie, *dialogic reflection*), 14 (3.4%) as level 3 (ie, *transformative reflection*), and 2 (0.5%) as level 4 (ie, *critical reflection*).

#### Reflection Level 0 (Description: Revisiting)

Responses that were rated as reflection level 0 were judged to be not reflective and often emphasized one’s desire or intention to increase one’s physical activity levels, simply repeated or paraphrased subject matter content, or provided commentary on the delivery of the subject matter itself. The themes we identified for reflection level 0 responses related to (1) *making a resolution*, (2) *knowledge of subject matter*, and (3) *appreciation or distaste for intervention content*.

Participants often made short imperative statements concerning a general need to increase physical activity or mindfulness (ie, *making a resolution*), but at this level of reflection, they did not demonstrate sufficient explanation to extend their response beyond merely revisiting intervention content (eg, “Just start!” [study identification number (SID) 148, 64 years]).

The theme related to *knowledge of subject matter* characterized a subset of responses that were descriptive paraphrases of didactic intervention content related to physical activity or ACT principles; for example, a participant wrote, “Even 10 minutes of activity is better than none” (SID 152, 54 years).

Responses that spoke to an *appreciation or distaste for intervention content* generally provided commentary on the intervention content without evidencing deeper reflection:

No real huge “ah ha” moments from this since it was all review. I had some problems with technology this time and got booted out twice before saving my answers so fewer long responses this week.

SID 103, 45 years

#### Reflection Level 1 (Reflective Description: Revisiting With Explanation)

Responses that were rated as reflection level 1 were judged to provide more justification than reflection level 0 comments, but this additional substance was generally descriptive in nature and without evidence of a deeper change in perspective. This reflection level made up the majority of responses (247/407, 60.7%). These comments commonly emphasized one’s desire or intention to increase one’s physical activity levels with some action-oriented or attitudinal elaboration. The themes we identified for reflection level 1 responses related to (1) *making a resolution*, (2) *personal application or action planning*, and (3) *kindness*.

*Making a resolution* was also a common theme for the level 1 responses. These responses were generally centered on the importance of increasing physical activity levels and provided a more nuanced explanation than the level 0 responses (eg, “[T]hat fitness in itself is a value that I should prioritize for overall happiness and well-being, not just when I want to lose weight and achieve a summer body” [SID 84, 32 years]).

Responses related to *personal application or action planning* tended to apply information from the intervention to the participant’s own life. This was often related to engaging in physical activity (eg, “One of the important takeaways for me is the proper way to stand and walk which allows our back to be straight...and assist with our posture” [SID 87, 64 years]). This theme also applied to using acceptance- and mindfulness-based techniques (eg, “Practical Action idea: Pausing to ‘unpack’ the negative thoughts, physically remove them” [SID 110, 74 years]).

Finally, the *kindness* theme captured responses that included an emphasis on being kind to oneself in the course of gradually increasing physical activity levels. This theme was also evident in comments that spoke to an appreciation of intervention content that normalized the reality of challenges experienced in the course of engaging in physical activity and cancer survivorship:

Life is hard and challenging and that is NORMAL. A rich full life is one that consists of a variety of emotions.

SID 93, 56 years

#### Reflection Level 2 (Dialogic Reflection: Exploring Relationships)

Responses that were rated as reflection level 2 were judged to exhibit reflection on more complex conceptual relationships than responses rated as reflection level 1. These responses often evidenced a change of perspective or a more nuanced consideration of the intervention content and its application to one’s own life. Reflection level 2 responses commonly displayed many of the themes presented previously (eg, *making a resolution* and *personal application or action planning*) but were further characterized by the themes of (1) *personal insight* or (2) *discussion of personal facilitators or mechanisms of behavior change*.

The theme of *personal insight* characterized comments that involved elements of personal introspection and reflection on oneself or one’s own thought and behavioral patterns:

It got me to thinking about what the negative thought might be. Why do I not want to exercise if I know it’s good for me? Somewhere way, way deep down I don’t think I believe it. That’s helpful to realize and good to see in print. Now to figure it out.

SID 120, 78 years

Other participants shared candid observations about the difficulties involved with satisfying perceived needs:

Need to refocus on what has been important, can be again but with physical limitations like a nagging injury, isolation due to the pandemic, restrictions where I live, too comfortable with aloneness now.

SID 139, 76 years

The theme of *discussion of personal facilitators or mechanisms of behavior change* applied to responses that evidenced reflection on what might help increase physical activity or how being more physically active might in turn support a valued aspect of the participant’s life:

I need to learn how to separate the thought from who I really am. I realize that I am overwhelmed by the “starting point,” that place where I am required to overcome the law of inertia in all the things I want or need to do. Once I get started I am generally able to complete the task.

SID 98, 53 years

#### Reflection Level 3 (Transformative Reflection: Fundamental Change)

This level of reflection was relatively rare (14/407, 3.4%). Responses determined to represent reflection level 3 were characterized by substantively deeper reflection than responses determined to represent reflection level 2—additionally marked by evidence suggestive of more profound *personal insight* and a fundamental *change in perspective*. In reflections that attained this level of depth, individuals often intimated that they revised personally held beliefs in ways that were empowering and conducive to enduring change:

I think about “the body keeps the score” and how some of the problems and thoughts and feelings I have are programmed into my DNA just like my propensity towards cancer. Some, but not all. I need to look at those moments where I am able to distract myself and notice the little victories. I’ll never be perfect—never have a perfect body a perfect kid a perfect life, but there are moments every day where I feel I’m doing it right and I need to notice those moments. Last week i ran 2 miles without stopping. Not much—I used to run 5 without stopping—but that was a great feeling. Last week I carved pumpkins with my son and my neighbor came over and we talked for over an hour. Thoughts of being unlovable and depressed faded during those moments. Also I’m trying to stay away from situations where I feel this loneliness and unlovable-ness... staying off the dating site I paid so much for hurts in one way but since nobody was calling me anyways it’s better just to not open the app up.

SID 104, 50 years

Participants evidencing this level of reflection often reframed challenges that related to their cancer journey or spoke about appreciating life from a different perspective:

I love the point just made: “Even if we fail at our goals, if we act in a way that’s consistent with our values we are successful.” I didn’t do as much walking this week, but I did add strength training this week and it’s amazing how much more I felt. And that’s not a typo. I don’t know how else to describe it than I just felt “more.”

SID 113, 50 years

#### Reflection Level 4 (Critical Reflection: Wider Implications)

Responses reaching reflection level 4 were very rare (2/407, 0.5%). These responses were judged to meet the level of intrapersonal depth characteristic of reflection level 3 as well as to incorporate some of the wider social and ethical contexts in which the participants’ experiences were situated. The responses spoke to a pressing tension the participants experienced as survivors of cancer in navigating social and community-level factors (1 quote is presented herein, and another quote is presented in Table 2):

Dr. Harris’ video was great to hear. When I was going through chemo some people would say, “Keep a positive attitude” and I didn’t always want to. I wanted to withdraw or rage sometimes. I think it’s easier for others to see you happy, it relieves their tension about what is happening to you. It does not mean you are not living a rich and fulfilling life and I was HAPPY to hear him say that.

SID 120, 78 years

### Intervention Adherence Predicting Outcomes

We observed associations between intervention adherence and change in 2 outcomes of interest (Table 3). Higher intervention adherence was associated with higher weekly bouts of muscle-strengthening physical activity at follow-up, adjusting for baseline levels, age, time since diagnosis, stage at diagnosis, race, ethnicity, education level, BMI category, and mean reflection level (B=0.26, SE 0.12, 95% CI 0.02-0.50; Cohen *d*=2.17). Participants in the fourth quartile of intervention adherence (21/75, 28%) averaged an increase of 0.38 bouts of muscle-strengthening physical activity in a typical week from baseline to follow-up, whereas those in the first quartile (40/75, 53%) averaged an increase of 1.43 bouts in a typical week. Similarly, higher intervention adherence was associated with less sleep disturbance at follow-up, adjusting for baseline levels and other covariates (B=−1.04, SE 0.50, 95% CI −2.02 to −0.06; Cohen *d*=2.08). Participants in the fourth quartile of intervention adherence averaged a decrease of 2.31 in the PROMIS-29 sleep disturbance score from baseline to follow-up, whereas those in the first quartile averaged a decrease of 4.68 in the PROMIS-29 sleep disturbance score. We did not observe statistically significant associations between intervention adherence and other outcomes of interest.

**Table 3:** Results from multiple linear regression analyses with independent variables regressed on intervention adherence and mean reflection level (n=75).

Outcome variable			Intervention adherence^a^		Mean reflection level^b^
			Estimate (SE; 95% CI)		Estimate (SE; 95% CI)
**Self-reported weekly physical activity**					
	Aerobic moderate to vigorous exercise (min)	11.75 (8.08; −4.09 to 27.59)		−30.84 (31.50; −92.58 to 30.90)	
	Muscle-strengthening exercise (bouts)	0.26 (0.12; 0.02 to 0.50)		0.11 (0.49; −0.85 to 1.07)	
**BREQ-3^c^**					
	Identified regulation	0.01 (0.04; −0.07 to 0.09)		0.06 (0.16; −0.25 to 0.37)	
	Integrated regulation	0.00 (0.06; −0.12 to 0.12)		0.55 (0.25; 0.06 to 1.04)	
	Intrinsic regulation	0.01 (0.06; −0.11 to 0.13)		0.28 (0.25; −0.21 to 0.77)	
**PAAQ^d^**					
	Cognitive acceptance	−0.07 (0.41; −0.87 to 0.73)		3.42 (1.70; 0.09 to 6.75)	
	Behavioral commitment	0.07 (0.32; −0.56 to 0.70)		0.93 (1.33; −1.68 to 3.54)	
**PROMIS-29^e^**					
	Physical functioning	0.37 (0.41; −0.43 to 1.17)		−1.77 (1.65; −5.00 to 1.46)	
	Anxiety	0.82 (0.49; −0.14 to 1.78)		−1.19 (2.07; −5.25 to 2.87)	
	Depressive symptoms	−0.03 (0.41; −0.83 to 0.77)		2.45 (1.72; −0.92 to 5.82)	
	Fatigue	−0.18 (0.65; −1.45 to 1.09)		0.84 (2.66; −4.37 to 6.05)	
	Sleep disturbance	−1.04 (0.50; −2.02 to −0.06)		1.16 (2.07; −2.90 to 5.22)	
	Social roles	−0.28 (0.37; −1.01 to 0.45)		−0.83 (1.54; −3.85 to 2.19)	
	Pain interference	0.47 (0.60; −0.71 to 1.65)		-0.12 (2.62; −5.26 to 5.02)	
	Pain intensity	0.11 (0.13; −0.14 to 0.36)		0.09 (0.58; −1.05 to 1.23)	

^a^Adjusting for age, time since diagnosis, stage at diagnosis, race, ethnicity, education level, BMI category, mean reflection level, and baseline value of the construct.

^b^Adjusting for age, time since diagnosis, stage at diagnosis, race, ethnicity, education level, BMI category, intervention adherence, and baseline value of the construct.

^c^BREQ-3: Behavioral Regulation for Exercise Questionnaire-3.

^d^PAAQ: Physical Activity Acceptance Questionnaire.

^e^PROMIS: Patient-Reported Outcomes Measurement Information System.

### Mean Level of Reflection Predicting Outcomes

We observed associations between participants’ mean reflection level and change in 2 outcomes of interest (Table 3). Higher mean reflection level was associated with higher integrated regulation at follow-up, adjusting for baseline levels, age, time since diagnosis, stage at diagnosis, race, ethnicity, education level, BMI category, and intervention adherence (B=0.55, SE 0.25, 95% CI 0.06-1.04; Cohen *d*=2.20). Similarly, higher mean reflection level was associated with higher cognitive acceptance of physical activity at follow-up, adjusting for baseline levels and all other covariates (B=3.42, SE 1.70, 95% CI 0.09-6.75; Cohen *d*=2.01). We did not observe statistically significant associations between mean reflection level and other outcomes of interest.

## Discussion

### Principal Findings

This study was a secondary analysis of data from a digitally mediated intervention centered on applying principles and techniques from ACT to help people living with and beyond breast cancer overcome internal barriers to physical activity (eg, pain, fatigue, and frustration). We applied the framework presented by Fleck and Fitzpatrick [40] to gauge the depth of reflection evident in participants’ written responses to prompts encouraging reflection on the intervention’s weekly modules. We conducted content analysis to characterize participants’ responses and multiple linear regression analyses to explore to what extent intervention adherence and participants’ mean level of reflection were associated with study outcomes (ie, physical activity behaviors, motivation for physical activity, physical activity acceptance, and health-related quality of life). There were 407 written responses from the 75 participants over the course of 7 modules. Most of the responses were rated as either reflection level 0 (ie, *description*; 70/407, 17.2%) or level 1 (ie, *reflective description*; 247/407, 60.7%). Of the 407 responses, 90 (22.1%) demonstrated evidence of deeper levels of reflection. Intervention adherence was associated with more muscle-strengthening physical activity and better sleep outcomes. Mean reflection level was associated with more integrated motivation for physical activity and higher willingness to experience the full range of sensations that may accompany physical activity.

### Comparison With Previous Literature

This study extends previous literature that has highlighted the importance of the cognitive and experiential aspects of DBCI engagement. To date, these aspects of DBCI engagement have largely been inferred from behavioral data (ie, system use metrics). In their review of methodologies for measuring engagement with DBCIs, Short et al [26] suggested that researchers should investigate how intervention content affects the cognitive and experiential aspects of DBCI engagement via the inclusion of open-ended items. We took this approach and found that participants’ responses to open-ended items could be evaluated using the reflection framework presented by Fleck and Fitzpatrick [40]. This approach is consistent with research in the education literature that has found reflective writing samples to be amenable to the quantitative assessment of the depth of reflection [35].

Our findings regarding the breakdown of reflection levels evident in this study were consistent with reviews that have evaluated reflective writing in the context of health-related education [39,47]. Even among graduate students, the great majority of reflective processes tend to occur at the descriptive level, and transformative and critical reflections are consistently rare [39,47]. There is limited literature applying these or similar techniques to better understand programs for health education and health promotion [39,47]. In this study, we observed considerable variation in the reflective depth of responses within and between individuals. This suggests that depth of reflection may be modifiable in this context. Indeed, reflection is a common goal of educational interventions, and it seems to be modifiable in other contexts [35]. Providing appropriate scaffolding, fostering collaboration in learning, and using varied exercises to stimulate reflection have been identified as techniques that may increase learners’ depth of reflection [35,39]. Time constraints, conflicting values, a lack of feedback, and a lack of trust have been identified as barriers to learning in the context of educational interventions promoting student reflection [35,39]. Future research is needed to identify how DBCIs might be designed to facilitate meaningful reflection and most effectively target the psychosocial determinants of lasting behavior change.

The themes identified in our qualitative analysis provided some insight into the variability of cognitive processes occurring in participants who experienced the digital physical activity intervention. Common themes in the lower levels of reflection generally suggested that participants had acquired new knowledge and endorsed intentions to change their physical activity behaviors (eg, *making a resolution* and *knowledge of subject matter*). These are important antecedents of successful behavior change [48]. However, they are generally not sufficient for realizing physical activity adherence goals; nearly half of the people who indicate that they intend to change their physical activity patterns do not do so [49]. The themes that characterized higher levels of reflective comments are concordant with processes that have been shown to moderate this *intention-behavior gap* [50]. *Discussion of personal facilitators or mechanisms of behavior change* and experiencing *personal insight* or a *fundamental change in perspective* conceptually align with the self-regulatory processes and notions of physical activity identity that tend to moderate the relationship between intention and physical activity adherence [50]. Encouraging personal reflection may be an autonomy-supportive approach to health promotion [34,51,52]. More research is needed to better understand if and how engaging in deep reflection may influence the conscious (eg, affective attitude) and automatic (eg, identity) processes that underlie successful behavior change.

Reflection may be a type of cognitive engagement uniquely suited to supporting knowledge transfer into other domains and contexts [36,53,54]. Schon [55] provides detailed commentary on reflective processes and distinguishes *reflection-on-action* (critical retrospective analysis) from *reflection-in-action* (conscious awareness of real-time behavioral modification). The author emphasizes the primacy of the latter as a determinant of sustained change and suggests that *reflection-on-action* may serve as a prerequisite for implementing change in real time. We found participants’ average depth of retrospective reflection to be associated with more integrated regulation for physical activity and cognitive acceptance in the context of physical activity but not with other outcomes of interest. It is encouraging that deeper levels of reflection on intervention content were associated with beneficial changes in motivation and physical activity acceptance, given that these were the theory-informed psychosocial constructs that the DBCI targeted [23]. The cognitive acceptance of physical activity has been shown to be associated with long-term changes in objectively measured physical activity, and integrated regulation is similarly predictive of physical activity [45,56]. However, in this study, we did not observe evidence that the depth of reflection was associated with physical activity–related outcomes or other outcomes of interest. Simple intervention adherence was associated with increases in muscle-strengthening physical activity and reductions in sleep disturbance. This may be due to the intervention’s inclusion of practical resources for muscle-strengthening physical activity and mindfulness exercises. Our findings suggest that designing DBCIs to encourage reflective processes may help target some theory-supported mechanisms of action but may not always be necessary to engender changes in desired end points. More research is needed to investigate whether deep reflection mediates changes in key long-term behavioral and health outcomes and to what degree it should be prioritized in DBCIs.

Considering the depth of reflection may be a useful lens through which to evaluate participants’ cognitive engagement with the didactic components of DBCIs. At present, DBCIs are commonly oriented toward maximizing participant engagement as measured by system use. However, rather than simply attempting to maximize DBCI system use, it may be beneficial to target aspects of *effective* engagement [29]. In this study, participants’ depth of reflection was associated with 2 key study outcomes. It may be possible to design dynamic interventions that optimize psychological processes such as critical reflection. This endeavor would be supported by the ability to derive metrics that reliably reflect underlying psychological processes from participants’ verbal output. We demonstrated that applying the reflection framework presented by Fleck and Fitzpatrick [40] is a promising approach to quantifying participants’ qualitative data. Emerging technologies such as large language models may be applied to this end to make this process more expedient and conducive to just-in-time adaptive interventions. Digital health promotion efforts centered on optimizing psychological processes such as critical reflection may supplement or supplant approaches that are narrowly oriented toward maximizing system use.

Reflective writing can serve as a means of self-expression and have therapeutic effects [57]. However, it can also be perceived as burdensome by some individuals. Perski et al [30] emphasized the role played by participants’ subjective experiences, characterized by attention, interest, and affect, in DBCI engagement. There are likely important trade-offs that occur in optimizing for cognitive versus affective aspects of DBCI engagement. Participants’ comments in this study suggested that although many enjoyed the introspective aspects of the intervention, some did not. This observation is concordant with high ratings of acceptability in the parent study for those who completed the study but a less-than-ideal dropout rate (23.7%) [23]. Achieving deeper levels of reflection may have a dynamic and, in some contexts, diametrically opposed relationship with affective or subjective experiences. Particularly given the importance of affect in physical activity behavior [58], future research should investigate the interrelationships among these aspects of DBCI engagement and how to strike the right balance for different individuals in different contexts.

### Limitations

The findings from this study must be considered in the context of its limitations. A small sample size, possible selection bias, and high attrition limit the generalizability of the findings of this study; the analytical sample was a convenience sample that was relatively well educated and had limited racial and ethnic diversity. Short et al [26] highlight that the use of qualitative methods to assess DBCI engagement is inherently limited by a lack of generalizability. The application of the reflection framework presented by Fleck and Fitzpatrick [40] along with directed content analytic methods may facilitate comparison in future studies; however, it is important to note that there may be other dimensions of reflection. We were primarily interested in assessing the vertical dimension of reflection (ie, the depth of reflection), but reflection has also been characterized as having an iterative process-oriented dimension that can be nonlinear and cyclic [39]. Given this prospect and the sometimes cyclical nature of behavior change itself [59], it may be beneficial to longitudinally investigate reflective processes and their bidirectional relationships with physical activity behaviors and determinants. Likewise, there are other ways to define intervention adherence. A limitation of this study is that we did not obtain other metrics that might characterize adherence, such as time spent in the modules. In future studies, obtaining more granular data concerning system use may be advantageous. Given that the study was conducted during the COVID-19 pandemic, history was a threat that may have influenced participants’ reflective processes and physical activity–related constructs. Reliance on the self-reported assessment of physical activity patterns also has well-documented limitations. Finally, we conducted multiple statistical tests and, although this investigation was explicitly exploratory in nature, the study findings are prone to an inflated chance of type I error.

### Conclusions

In this study, we sought to explore a novel method for understanding critical reflection occurring in a DBCI designed to increase physical activity in insufficiently active people living with and beyond breast cancer. We found the application of qualitative content analysis based on the reflection framework presented by Fleck and Fitzpatrick [40] to be a useful tool for helping to gauge the extent to which participants engaged in reflective processes. Furthermore, we found that deeper reflection levels tended to be associated with changes in the targeted psychosocial constructs. Reflecting on newly acquired information is a critical process in integrating relevant insights for sustained behavior change. Encouraging personal reflection is an autonomy-supportive approach to promoting physical activity, and more research is warranted to investigate this approach in DBCIs serving people living with and beyond cancer.

## Abbreviations

ACT: acceptance and commitment therapy
BREQ-3: Behavioral Regulation for Exercise Questionnaire-3
DBCI: digital behavior change intervention
PAAQ: Physical Activity Acceptance Questionnaire
PROMIS: Patient-Reported Outcomes Measurement Information System
REDCap: Research Electronic Data Capture
SID: study identification number
SDT: self-determination theory
